# Unveiling Cardiovascular Outcomes: A Comparative Analysis of CABG Recipients versus Non-CABG Patients in the Management of Acute Coronary Syndrome (ACS)

**DOI:** 10.31661/gmj.v13i.3260

**Published:** 2024-06-27

**Authors:** Naser Aslanabadi, Ahmad Separham, Hormoz Golshani, Elnaz Javanshir, Razieh Parizad, Ahmad Ahmadzadehpournaky

**Affiliations:** ^1^ Cardiovascular Research Center, Tabriz University of Medical Sciences, Tabriz, Iran

**Keywords:** Acute Coronary Syndrome, Coronary Balloon Angioplasty, Coronary Artery Bypass Grafting

## Abstract

Background: The history of bypass surgery for coronary arteries and subsequent coronary angioplasty is a crucial and vital issue for patients with acute coronary syndrome (ACS). This study aims to investigate and compare the occurrence of cardiovascular events in patients with a history of Coronary Artery Bypass Grafting (CABG) versus those without such a history, specifically focusing on individuals diagnosed with ACS. Materials and Methods: This cohort study was conducted at Madani Hospital in Tabriz, Iran. Patients diagnosed with ACS who were hospitalized and underwent Percutaneous Coronary Intervention (PCI) from the beginning of 2018 to the beginning of 2020 were included. The records for follow-up regarding mortality and cardiovascular events were documented for the next three years (2020 to 2023). Subsequently, patients were categorized into two groups: those with a history of CABG and those without a history of CABG. Patients of each study group were divided into two groups: ST-segment elevation acute coronary syndrome (STEA)CS/primary PCI and non-ST-segment elevation acute coronary syndrome (NSTEACS)/PCI, a total of approximately 473 cases were collected. The study groups were compared in terms of in-hospital and long-term cardiovascular events as well as other clinical outcomes. Results: A comparison of hospital and long-term events between the CABG group and the control group demonstrated a significant difference only in cases of recurrent myocardial infarction (MI)/ACS in long-term events (P=0.001). Additionally, comparing hospital and long-term events in the CABG group and the STEACS/NSTEACS control group revealed a significant difference only in cases of recurrent MI/ACS in long-term events (P=0.05). Conclusion: Patients with a history of CABG may face a higher risk of cardiovascular events, especially in recurrent MI/ACS. A thorough examination and closer monitoring of this patient group are needed to ensure improvement and mitigate the risks associated with potential complications arising from previous CABG surgeries.

## Introduction

Acute Coronary Syndrome (ACS) is a complex and serious condition in cardiovascular events that occurs due to a reduction or interruption of blood flow to the heart muscle [1]. ACS encompasses two primary conditions. Non-ST-segment Elevation Acute Coronary Syndrome (NSTEACS) manifests abruptly during minimal physical activity or at rest, inducing stable angina and Unstable Angina (UA). Additionally, ST-segment Elevation Myocardial Infarction (STEACS) arises from the sudden complete occlusion of a coronary artery, leading to damage in the heart muscle [2][3]. The prevalence of cardiovascular diseases (CVD), coronary artery diseases (CAD), and cardiovascular events is increasing worldwide and in Iran [4]. In the 2016 report of the American Heart Association (AHA), it was stated that 5.15 million people in the United States aged 20 and older are affected by CAD [5]. In 2021, 375,476 individuals lost their lives due to CAD. Approximately 1 in 20 adults aged 20 and above are affected by CAD (about 5%). In 2019, 5.5% of adults reported being diagnosed with CVD [6]. Although there are various methods for diagnosing this disease, angiography is still considered the gold standard. Coronary angioplasty is a therapeutic method used in the treatment of CAD. On the other hand, for half a century, revascularization of arteries has been the main treatment for CAD. The most common type of revascularization is open-heart surgery or Coronary Artery Bypass Grafting (CABG) [7].

Despite advances in secondary prevention measures in patients with a history of CABG, increased atherosclerosis in coronary arteries and rapid narrowing in saphenous vein grafts (SVGs) are of concern [8]. Follow-up studies aided by serial angiographies have shown that nearly 10% of SVGs experience occlusion in the first year, followed by continuous deterioration, which accelerates over time with the increasing lifespan of the graft [9]. Deterioration of these grafts is at least 2 to 5% per year. For this reason, ACS or myocardial infarction (MI) with ST-segment elevation due to obstruction in other coronary arteries or transplanted arteries is more common in patients with a history of CABG.

Understanding the implications of ACS in patients with a history of CABG undergoing coronary angioplasty is crucial due to the inherent complexities introduced by the previous surgical intervention. The study provides a comprehensive analysis of both short-term and long-term outcomes, shedding light on the potential risks and challenges faced by this specific patient population. Moreover, the study’s location in Iran, adds geographical diversity to the existing body of literature, potentially uncovering insights that may be influenced by regional variations in healthcare practices, patient demographics, and environmental factors. Therefore, this study aims to investigate and compare the occurrence of cardiovascular events in patients with a history of CABG versus those without such a history, specifically focusing on individuals diagnosed with ACS.

## Materials and Methods

**Figure-1:**
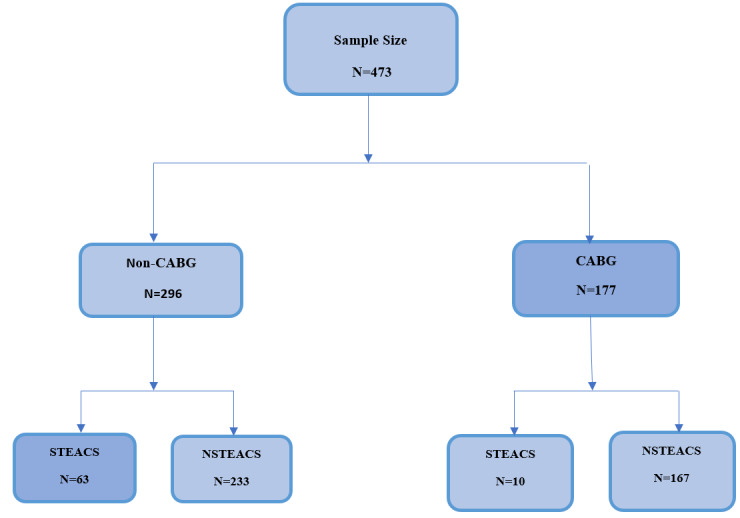
Figure 1. Classification diagram of the studied samples.

The present study was a retrospective cohort study that was performed in Shahid Madani Hospital, Tabriz, Iran, with a population coverage of about 200,000 people after approval by the ethics committee of Tabriz University of Medical Sciences with the code of ethics. (IR.TABZMED.REC.1402.087). The target community in this study included patients diagnosed with ACS admitted to -Madani Hospital from the beginning of 2018 to the beginning of the year 2020 and underwent Percutaneous Coronary Intervention (PCI). In the next 3 years (2020-2023), their documented files were also registered for follow-up on mortality and cardiovascular events. The exclusion criteria included incomplete file information, non-ACS-related visits, receiving thrombolytic therapy, not undergoing angioplasty due to inappropriate coronary anatomy, and lack of documented records for long-term complications in the 3 years following angioplasty.

In this study, patients were enrolled through a complete enumeration method. Subsequently, the patients were divided into two groups based on their history of CABG: those with a history of CABG and those without a history of CABG (control group). These two groups were then examined for hospital and long-term cardiovascular complications. ACS in these two patient groups was further categorized into two subgroups: STEACS/primary PCI and NSTEACS/PCI (Figure-1). The sampling was done through a complete enumeration method, and approximately 473 files were collected for analysis.

This study included a researcher-made questionnaire covering the demographic and clinical information of the patients. Demographic information comprised age, gender, and history of coronary artery disease risk factors. Clinical data included ischemia time, blood pressure (BP) upon admission, heart rate (HR) upon admission, territory of MI, KILLIP class, CABG information including the number and type of grafts (vein or artery) and run-off, angiographic details such as the type of dilatable angioplasty vessel (graft or native vessel), TIMI flow base, Post PCI TIMI flow, size and number of deployed stents, use of thrombectomy devices, GPIIbIIIa inhibitor usage, balloon pump usage, and distal protection device usage. Then, hospital and long-term complications were recorded. The data for this research were obtained from the hospitalization file of patients ACS patients meeting the study criteria at Madani Hospital in Tabriz, Iran. In this study, a checklist of patient file information, including demographic details, medical history, cardiovascular risk factors, clinical examinations, diagnostic tests, performed treatments, and hospital complications at the time of patient admission and during the hospitalization period, was systematically recorded. Subsequently, using the same checklist, information related to long-term complications during the three years of the patient’s subsequent visits, which were documented in continuous patient files, was considered. The information from these patients was collected after obtaining ethical committee approval and following ethical principles and medical confidentiality by the responsible physician. Finally, the recorded information in the checklist was evaluated and analyzed.

Statistical Analysis

Data analysis was done using SPSS version 25 (SPSS INC., IBM Corporation, Chicago, IL) software and the significance level of P<0.005 was considered significant. Quantitative data were presented as mean (± standard deviation), and qualitative data were presented as percentages and frequencies. Student’s t-test was used to describe quantitative variables and chi-square test was used for qualitative variables. The normality of data distribution was also evaluated using the Kolmogorov-Smirnov test. Long-term mortality was compared using the Kaplan-Meier method and the Log-rank test. To determine the independent value of CABG on hospital and long-term complications, Multivariate Regression Analysis was utilized. Confounding variables known to impact mortality were included in this model and the independent effect of CABG on in-hospital mortality and long-term major adverse cardiac events (MACE) was identified.

## Results

**Table:** Table1. Demographic Variables in Two Groups of CABG and Non-CABG

**Variables**	**Total**		**CABG**		**Non- CABG**		**P-value**
	Mean	SD	Mean	SD	Mean	SD	
Age	64.02	10.14	63.03	8.12	64.61	11.14	0.138 b
HB	14.29	1.97	13.48	1.58	14.56	1.90	<0.001 a
PLT	248.59	133.70	252.98	58.31	245.92	163.32	0.009 b
Cr	1.24	0.44	1.33	0.53	1.19	030	<0.136 b
Peak CTNI	2.01	0.42	1.88	0.53	2.75	0.52	0.012 b
Peak CKmb	41.79	22.14	27.79	17.14	51.42	25.56	<0.001 b
Bp on arrival	139.02	26.00	147.64	25.58	133.96	24.93	<0.001 b
HR on arrival	76.68	9.64	74.95	6.13	77.69	11.08	<0.001 b
LVEF on arrival	45.70	18.55	41.72	7.86	47.94	22.13	<0.001 b
Length of hospitalization	5.48	2.70	5.91	2.84	5.24	2.59	0.001 b
Door.To.Balloon (minutes)	57.30	24.26	92.48	62.05	51.28	38.85	0.001 b
Total.Ischemic (hours)	9.60	4.28	5.24	2.48	10.45	8.86	0.009 b

**a:** Independent-t test, **b:** Mann-Whitney U test P<0.05 considered as statistically significant. **HB:** Hemoglobin;**PLT:** Platelet Count; **Cr:** Creatinine; **CTNI:** cardiac troponin T; **BP:** Blood Pressure ; **HR:** Heart Rate; **LVEF:** Left ventricular ejection fraction; **CABG:** coronary artery bypass graft; **PCI:** Percutaneous Coronary Intervention

Results revealed that the average age of patients in the CABG group was approximately 63.03 years, while in the non- CABG, it was around 64.61 years. The average duration of Door-to-ballon in the CABG group and the non-CABG was 92.48 and 51.28 minutes, respectively, and there was a significant difference (P-value=0.001). The average duration of cardiac ischemia in the CABG group was significantly lower than the non-CABG patients (P-value=0.009).

The examination of demographic variables in both the CABG and non-CABG is presented in Table-1.

A comparison of hospital events and long-term events in the CABG group and the non-CABG showed no significant difference in cardiovascular events (p>0.05). However, a significant difference was observed between the CABG group and the non-CABG for recurrent ACS in long-term events (P=0.001, Table-2).

Comparison of hospital events and long-term events in the CABG group and the non-CABG for STEACS and NSTEACS showed a significant difference in terms of recurrent ACS in the long term (P=0.005). However, differences were not significant for other events (in-hospital death, CHF, stroke) (P>0.05, Table-3).

## Discussion

**Table:** Table2. Comparison of Hospital Events and Long-Term Events in the CABG and the non-CABG Groups

Variables		CABG		Non-CABG		P-value	P-value
N		%	N	%			
	Death	2	1.12	4	1.35	>0.999 b	
Hospital Events	CHF	4	2.25	6	2.02	>0.999 b	0.839
	Stroke	3	1.69	3	1.01	0.676 b	
	Re- ACS	0	0.0	0	0.0	>0.999 b	
	Death	0	0.0	1	0.33	>0.999 b	
Long-Term Events	CHF	3	1.69	6	2.02	>0.999 b	0.415
	Re- /ACS	70	39.54	73	24.66	0.001 a	

**CHF:** Congestive heart failure; **MI:** Myocardial infraction; **ACS:** Acute Coronary Syndrome, **CABG:** coronary artery bypass graft. P<0.05 is considered statistically significant **a:** Pearson Chi-Square, **b:** Fisher,s Exact test

**Table:** Table3. Comparison of Hospital Events and Long-Term Events in the CABG and the non-CABG Groups for STEACS and NSTEACS

					STEACS		NSTEACS				
		CABG			Non-CABG	P	CABG			Non- CABG	P
		N	%	N	%		N	%	N	%	
	Death	2	20.0	3	4.76	0.135 b	0	0.0%	1	0.42	>0.999 b
Hospital Events	CHF	0	0.0%	1	1.58	>0.999 b	4	2.39	5	2.14	>0.999 b
	Stroke	0	0.0%	2	3.17	>0.999 b	3	1.79	1	0.43	0.313 b
	Re- ACS	0	0.0%	0	0.00%	NA	0	0.0%	0	0.0%	>0.999 b
	Death	0	0.0%	0	0.00%	NA	0	0.0%	1	0.42	>0.999 b
Long-Term Events	CHF	0	0.0%	2	3.17	>0.999 b	3	1.79	4	1.71	>0.999 b
	Re- ACS	4	40.0	12	19.04	0.211 b	66	39.52	61	26.18	0.005 a

A Pearson Chi-Square, b Fisher’s Exact test **CABG:** Coronary artery bypass grafting; **CHF:** Congestive heart failure; **MI:** Myocardial infraction; **ACS:** Acute Coronary Syndrome, **CABG:** coronary artery bypass graft P<0.05 was considered significant. **a:** Pearson Chi-Square, **b:** Fisher,s Exact test

The present study was conducted with the purpose of clinical outcomes of patients with a history of CABG, referred with acute coronary syndrome, and undergoing coronary angioplasty. The results of the present study showed that the rate of hospital events of ACS patients who underwent PCI is not significantly different in patients with a history of CABG and patients without a history of CABG. A possible explanation for the lack of significant difference could be that the two groups (with and without a history of CABG) have similar baseline characteristics. If patients in both groups are similar in terms of age, comorbidities, the severity of CAD, and other relevant factors, it may contribute to the observed lack of differences in-hospital mortality. On the other hand, the PCI method may be equally effective in both groups. If PCI intervention is successful in restoring blood flow and managing CAD in both groups, it can lead to comparable outcomes in terms of hospital mortality. Additionally, post-PCI care and management of patients after the procedure may be similar in both groups.

If there is consistency in the quality of care, including monitoring and response to complications, stability can be achieved, contributing to similar hospital mortality outcomes [10].

Furthermore, advances in medical care and technology over time may minimize the impact of a history of CABG on hospital mortality in ACS patients undergoing PCI. Improved techniques and medical management can contribute to similar outcomes regardless of CABG history [11]. In this regard, the results of a study showed no significant difference in the mortality rate over time, at 30 days and one year post-hospitalization, between ACS patients with and without a history of CABG [12]. In the study of Iqbal et al. (2016) with the aim of investigating the outcome of PPCI intervention in patients with a previous history of CABG [13]. Louise P. Kohl et al.’s study (2014) with the aim of investigating the outcome of PPCI intervention in patients with a previous history of CABG showed that one-year events including mortality and cardiovascular complications were not different in both groups with and without a history of CABG, but five-year events in the group had more history of CABG [14].

The results of the present study indicate that the long-term mortality rate of ACS patients undergoing PCI does not show a significant difference between patients with a history of CABG and those without CABG history. In explaining this finding, it can be stated that patients in this study with and without a history of CABG have similar baseline characteristics, comorbidities, and risk factors, which may contribute to the observed lack of significant differences in long-term mortality. On the other hand, advancements in PCI techniques and technologies over time may have reduced the gap in outcomes between patients with and without a history of CABG. Improvements in stent technology and postoperative care may contribute to similar long-term results in both groups. The effectiveness of PCI in achieving optimal revascularization in patients with a history of CABG may be comparable to those without previous CABG. If both groups achieve similar levels of success in revascularization, it can lead to comparable long-term outcomes. The results of the current study indicate that there was a significant difference only in long-term events related to recurrent ACS between the CABG group and the non-CABG. Maybe, differentiation in ACS is important to consider when assessing the risk of recurrent events in individuals with a history of CABG. Differentiation in the level of ACS may reflect the progression of CAD over time. Even after CABG, patients may still have cardiovascular risk factors and disease progression, which can contribute to the occurrence of new ischemic events in the long term [15].

However, no significant differences were observed in other hospital and long-term events (in-hospital mortality, CHF, stroke). Possible reasons for this non-significant difference could be attributed to the benefits of CABG in improving blood flow to the heart muscle, reducing the risk of coronary artery disease progression, and enhancing overall cardiac function [16]. Nevertheless, both groups may share similar demographic characteristics that have influenced the occurrence of these events, challenging the attribution of differences solely to undergoing CABG.On the other hand, CABG can be particularly beneficial in the long term as it aids in managing the disease and reducing the likelihood of future cardiac events. Additionally, CABG may stabilize plaque accumulation in coronary arteries. Unstable plaques are more prone to rupture, leading to acute events like MI. By providing a new pathway for blood flow, CABG can reduce stress on existing plaques and potentially make them more stable [17].

Teixeira and colleagues reported no significant impact of previous CABG on short-term or midterm outcomes, such as mortality and undesirable cardiac events, in patients presenting with ACS [18]. Patients with previous CABG may have short-term outcomes similar to other patients but may experience more recurrent ischemic events during longer follow-up periods.

The results of this study illustrated that there was no significant difference in hospital events (death, CHF, Stroke, and ACS again) and long-term events of death and CHF between the group with and without CABG history in both NSTEACS and STEACS categories, but in the case of MI recurrent ACS in long-term events was different in the two groups of NSTEACS and STEACS, so that in STEACS patients there was no significant difference between these two groups with and without CABG history, but in NSTEACS patients a significant difference was observed between these two groups. The risk of subsequent ACS events over a long period is influenced by whether the patient has had NSTEACS or STEACS. On the other hand, the underlying pathophysiology of NSTEACS and STEACS is different. STEACS is often associated with complete coronary artery occlusion, whereas NSTEACS may involve partial occlusion [19]. These differences may contribute to changes in long-term outcomes and risk of complications.

In this regard, Elbarasi and colleagues demonstrated lower in-hospital mortality rates for patients with previous CABG in NSTE-ACS (1.7%) and previous CABG with PCI (0.9%) compared to patients without previous CABG (2.3%) [20]. Additionally, Kim and colleagues concluded that in-hospital mortality did not significantly differ in non-CABG patients with NSTEMI [21]. Mahmoud et al.’s study (2022) showed that patients with previous ACS and CABG usually present as unstable angina much less often than NSTEMI and rarely as STEMI and most events occur after one year [22].

This study focuses on the clinical outcomes of a specific and critically relevant subgroup of patients, those with a history of CABG who subsequently experience ACS and undergo coronary angioplasty. While various studies have explored outcomes in ACS or post-CABG scenarios independently, this research uniquely bridges the gap, delving into the intricate interplay between these two significant cardiac interventions. In addition, the three-year follow-up period further contributes to the novelty, allowing for a robust evaluation of the sustained impact of the interventions on patient health.

One of the limitations of this study is that the selected sample is solely from patients at Madani Hospital in Tabriz, Iran which may restrict the generalizability of the results to the broader population. Furthermore, the information has been collected based on a systematic patient registration program, but it’s possible that some crucial details or data might have been overlooked, potentially influencing the obtained results.

## Conclusion

The results of the study showed that during hospitalization, there was no significant difference in the occurrence of hospital events (death, stroke, CHF, recurrent ACS) between patients with a history of CABG and those without a history of CABG, both in the overall comparison of the two groups and in the separate comparison in the NSTEACS and STEACS groups.

Also, in the investigation of long-term events (death, stroke, CHF, recurrent ACS) in a three-year period, there was no significant difference in the rate of death and CHF between patients with a history of CABG and those without a history of CABG, both in the overall comparison of the two groups and in the separate comparison in the NSTEACS and STEACS groups. However, patients with a history of CABG significantly developed more ACS than patients without a history of CABG, and when these two groups of patients were compared in the NSTEACS and STEACS groups, this difference was not significant in the STEACS group, but it was significant in the NSTEACS group.

## Acknowledgment

This research was a cross-sectional study approved by the ethics committee of Tabriz University of Medical Sciences. We thank all the patients who participated in this study.

## Conflict of interest

None.
